# Effectiveness and Complications of Ultrasound Guided Fine Needle Aspiration for Primary Liver Cancer in a Chinese Population with Serum α-Fetoprotein Levels ≤200 ng/ml - A Study Based on 4,312 Patients

**DOI:** 10.1371/journal.pone.0101536

**Published:** 2014-08-29

**Authors:** Qi-wen Chen, Chien-shan Cheng, Hao Chen, Zhou-yu Ning, Shi-feng Tang, Xun Zhang, Xiao-yan Zhu, Sonya Vargulick, Ye-hua Shen, Yong-qiang Hua, Jing Xie, Wei-dong Shi, Hui-feng Gao, Li-tao Xu, Lan-yun Feng, Jun-hua Lin, Zhen Chen, Lu-ming Liu, Bo Ping, Zhi-qiang Meng

**Affiliations:** 1 Department of Integrative Oncology, Fudan University Shanghai Cancer Center, Shanghai, China, Department of Oncology, Shanghai Medical College, Fudan University, Shanghai, China; 2 School of Chinese Medicine, the University of Hong Kong, Pokfulam, Hong Kong, China; 3 First Department, Cancer Treatment Center, Weifang Hospital of Traditional Chinese Medicine, Weifang, Shandong Province, China; 4 Department of Ultrasound, Fudan University Shanghai Cancer Center, Shanghai, China, Department of Oncology, Shanghai Medical College, Fudan University, Shanghai, China; 5 Albany College of Pharmacy and Health Sciences, Albany, New York, United States of America; 6 Department of Pathology, Fudan University Shanghai Cancer Center, Shanghai, China, Department of Oncology, Shanghai Medical College, Fudan University, Shanghai, China; University of Pisa, Italy

## Abstract

**Background:**

Hepatocellular carcinoma (HCC) can be diagnosed by noninvasive approaches with serum α-fetoprotein (AFP) levels >200 ng/ml and/or a radiological imaging study of tumor mass >2 cm in patients with chronic liver disease. Percutaneous fine needle aspiration (FNA) under ultrasound (US) guidance has a diagnostic specificity of 95% and is superior to radiological imaging studies.

**Aim:**

The aim of this study is to elucidate the effectiveness and complications of fine needle aspiration in a Chinese population with primary liver cancer and AFP levels ≤200 ng/ml.

**Materials and Methods:**

A retrospective study was conducted over a period of 28 years. This selection period included patients with a suspected diagnosis of primary liver cancer whose AFP levels were ≤200 ng/ml and who underwent US-FNA. This data was then analyzed with cytomorphological features correlating with medical history, radiological imaging, AFP, and follow-up information.

**Results:**

Of the 1,929 cases with AFP ≤200 mg/ml, 1,756 underwent FNA. Of these, 1,590 cases were determined malignant and the remaining 166 were determined benign. Further, 1,478 malignant cases were diagnosed by FNA alone, and of these, 1,138 were diagnosed as PLC. The sensitivity, specificity, positive predictive value, negative predictive value, and overall accuracy of the diagnoses were 92.96%, 100%, 100%, 59.71%, and 93.62% respectively. There was no significant difference in the sensitivity, specificity, PPV and NPV between the subgroups with tumor size<2 cm and ≥2 cm. Major complications included implantation metastasis and hemorrhage.

**Conclusion:**

Patients with PLC, especially those who present with an AFP ≤200 ng/ml, should undergo FNA. If negative results are obtained by FNA, it still could be HCC and repeated FNA procedure may be needed if highly suspicious of HCC on imaging study. The superiority of FNA in overall accuracy may outweigh its potential complications, such like hemorrhage and implantation metastasis.

## Introduction

### Primary Liver Cancer

Primary Liver Cancer (PLC) is one of the most common malignant neoplasms in the world and its incidence has risen as a result of the increased global burden of liver disease. China, which is a hepatitis B pandemic area, is also a hepatocellular carcinoma (HCC) high-incidence region, where 55% of the world's HCC occurs [1]. As a result of rather asymptomatic features, most initial diagnoses of HCC are at late stage and characterized by large tumor size and unresectable lesions, in combination with poor liver function, ultimately leading to a shortened life expectancy. Therefore, disease surveillance is not only important in high risk patients for early diagnosis, but also to have accurate diagnostic methods for further appropriate treatments.

### Serum α-fetoprotein Level

Alpha-fetoprotein (AFP), a serum glycoprotein physiologically produced by the fetal liver and yolk sac, was the first recognized and is the most commonly used marker for the detection of HCC. An elevated serum AFP level may be physiologically seen in pregnancy and pathologically seen in tumors of gonadal origin, hepatoid adenocarcinoma, HCC, or nonmalignant chronic liver diseases, including acute or chronic viral hepatitis [2]. AFP is assumed to be more indicative of non-hepatitis virus infected patients in terms of HCC. According to the 2005 American Association for the Study of Liver Diseases (AASLD) practice guidelines [3], the noninvasive diagnostic approach of HCC should be based on the consideration of serum α-fetoprotein levels >200 ng/ml and/or a radiological imaging displaying tumor masses >2 cm in patients with chronic liver disease. Besides following the AASLD guidelines, the decision to use AFP >200 ng/ml as a cut-off point in this retrospective study was based on a case-control study reported by Trevisani, et al. [4] in 2001. Trevisani, et al. reported that AFP>200 ng/ml has a substantial sensitivity and specificity for not only diagnostic and confirmatory testing for HCC, but also in a cost-effective investigation that is optimal for HCC screening. Despite the specificity of AFP, up to 40% of small HCC patients present with normal AFP levels at initial diagnosis, whereas 30% of HCC patients present with unelevated serum AFP levels [5]. As a result, other investigations such as fine needle aspiration (FNA) and radiological imaging should be used in combination with AFP levels for HCC diagnosis.

### Fine Needle Aspiration

Percutaneous FNA, a minimally invasive approach used to establish the diagnosis of HCC, can obtain both cytological and histological samples via ultrasound (US) or CT guidance. Although advancements in imaging techniques have occurred in recent years, the diagnostic value of FNA should still be used for optimal sensitivity and specificity, as well as, for future guidance and determination of chemotherapy regimens and other treatment approaches. Even if the AASLD guidelines are followed closely using AFP >200 ng/ml as a diagnostic standard, there is still a possibility of a false positive diagnosis, which may result in unnecessary treatments and suffering. The possibility of a false negative diagnosis made by false interpretation of low serum AFP, should not be neglected either. Therefore, in this study, we aimed to reevaluate the significance of serum AFP ≤200 ng/ml in this patient population as a diagnostic value in the application of US-FNA to determine the overall accuracy of this procedure and to guide future clinical practice.

## Materials and Methods

### Patient characteristics

This study was approved by the ethics committee of Fudan University Shanghai Cancer Center. The retrospective study covered a period of 28 years, from June 1985 to December 2012. We retrospectively analyzed the medical records of 4,312 patients suspected of PLC who were admitted to Fudan University Shanghai Cancer Center, Shanghai, China. Of the patients recommended for FNA, 188 were excluded from this study due to loss of follow-up. Among the remaining patients, 1,929 presented with serum AFP levels ≤200 ng/ml. Of these, 1,756 underwent FNA, and 173 were excluded due to FNA failure. Patient characteristics of the 1,756 cases who underwent FNA are presented in Table 1.

**Table 1:** Patient characteristics of the 1,756 cases that underwent FNA.

Patient characteristics	No. of Cases
Gender	
Male	1459(83.09%)
Female	297(16.91%)
AFP Level	
<50 ng/ml	466(26.54%)
50–100 ng/ml	355(20.22%)
100–150 ng/ml	377(21.47%)
150–200 ng/ml	558(31.78%)
Tumor Size	
<20 mm	149(8.49%)
20–50 mm	209(11.90%)
50–80 mm	572(32.57%)
80–100 mm	474(26.99%)
>100 mm	352(20.05%)
Liver Cirrhosis	
Yes	932(53.08%)
No	824(46.92%)
HBsAg Status	
Positive	1038(59.11%)
Negetive	718(40.89%)

### US-FNA technique

Before the procedure, written informed consent was obtained from all patients. FNA was aborted if prothrombin time was prolonged over 5 seconds and/or if platelet counts fell below 50,000/µl. The procedures were performed under local anesthesia in the intervention room by a group of experienced oncologists, US specialized radiology technicians, and a cytotechnologist. Under ultrasonographic guidance, a safe needle pathway was determined to avoid intraparenchymal vessels and biliary structures. Tumors located near the hepatic capsule and diaphragm were avoided to prevent internal hemorrhage and diapharmatic motion. An 18-gauge guiding needle was used to aim a 22-gauge Chiba needle at the suspected lession. The Chiba needle was inserted coaxially through the guide needle into the mass with careful guidance by US imaging.

Under negative internal pressure, the needle was moved up and down within the tumor mass two to three times and cells were extracted. With the presence of a cytotechnologist, the adequacy of the sample collection was ensured to avoid failure of pathocytological findings due to lack of sample size. After FNA, the smears were fixed immediately, taken to the laboratory for H&E staining, and the final cytological diagnosis was typically made within two days. Immediately after FNA, an US examination was performed to detect any abnormalities or complications, such as intraperitoneal hemorrhage. Manual compression was made at the puncture site and a compressive bandage with a compression bag was positioned appropriately. Vital signs, including heart rate, blood pressure, respiratory frequency, blood oxygen saturation, and body temperature were monitored for 6 hours after US-FNA. Over the last five years, anhydrous ethanol has been applied to the wound as routine practice after needle removal.

### Criteria for final diagnosis

In our study, final diagnosis was determined according to histopathological or initial cytological findings of malignancy, combined with imaging or clinical follow-up outcomes, including disease progression or death due to the disease. The term “primary liver cancer (PLC)” was used as a final diagnosis as most of the patients were not eligible to receive surgery or could not be further classified into a specific histopathological type. The definite diagnosis of PLC was confirmed either by post-surgical histopathological findings or positive radiological evidence, and/or increased serum AFP levels, combined with disease progression or death in clinical follow-up. Initial malignant cytological findings with positive follow-up radiological findings contributed to the final diagnosis.

### Statistical analysis

Standard methods were used and test performance was determined by calculating sensitivity, specificity, positive predictive value (PPV), negative predictive value (NPV), and overall diagnostic accuracy compared to the final diagnosis after follow-up. A chi-square test was used to compare the difference between the two groups. And P value<.05 was considered as statistically different. Cytomorphological investigation revealed atypical lesions, as well as, suspicious and unsatisfactory cases that were later diagnosed as malignancies by either clinical imaging and/or follow-up (wait-and-see). These cases were deemed as false negatives in the study.

## Results

### US-FNA failure cases

Of the 1,929 patients, 173 failed FNA. The most common reason for FNA failure was due to tumor location near the surface of the liver capsule or near the diaphragm, which accounted for 113 cases. Eighteen patients failed FNA and were excluded because of abnormal coagulation indexes that increased the risk of internal bleeding and could not be corrected within the short time period. Abnormal coagulation indexes included prolonged prothrombin time (>5 s), thrombocytopenia (platelet count <50,000/µl), or elevated INR >2. The procedure was not performed in six patients who failed to provide written informed consent. Infiltrative type tumors were detected in eleven patients, in which definite focal lesions could not be found. Only a small portion of failure cases (25 patients) had a contraindication for FNA. Contraindications included: low performance status (KPS<70), ascites, internal tumor liquefaction necrosis, significantly elevated total bilirubin possibly due to extrahepatic biliary obstruction and highly obstructive jaundice, severe congestive heart failure, severe infection, uncooperative patients, or coma.

### Diagnostic outcome

Overall, a positive diagnosis of malignancy was confirmed in 1,478 cases, which were subclassified based on cytomorphology into tumor types. The cytological diagnoses classified as definitively malignant on aspirate material are shown in Table 2 and Table 3. Of the 1,478 cases, 1,138 were diagnosed as PLC. Twenty-three cases reported inconclusive cytology results, including fifteen cases suspicious for malignancy and eight cases containing atypical cells. Twelve cases of the suspicious group and four cases of the atypical cell group were later confirmed malignant and determined PLC. Initially, a total of 251 cases were reported negative for malignancy, however, on clinical follow-up or post-operative pathology, 92 were proven malignant. The diagnosis included 71 PLC, 19 metastatic cancer, and two sarcoma cases. There were also four additional cases in which necrotic tissue with no evidence of malignancy originally was later proven malignant by clinical and imaging follow-up. Serum AFP levels were used to analyze the sensitivity, specificity, PPV, NPV, and diagnostic accuracy of FNA (Table 4). Meanwhile, there was no significant difference in the sensitivity, specificity, PPV and NPV according to P value between the subgroups with tumor size<2 cm and ≥2 cm (Table 5).

**Table 2:** Cytological diagnoses for 1,478 cases as malignant.

Final Diagnosis	No. of Cases
Primary liver neoplasm	1145
HCC	1067
CCA	63
HCC+CCA	8
Hepatoblastoma	1
Lymphoma	6
Metastatic Neoplasms	75
Adenocarcinoma	68
Squamouscell carcinoma	7
Malignancy NOS	258
Carcinoma	142
Adenocarcinoma	94
Sarcoma	21
Carcinosarcoma	1
Total	1478

NOS, not otherwise specified; HCC, hepatocellular carcinoma; CCA, cholangiocarcinoma; HCC+CCC, Mixed hepatocellular and cholangiocarcinoma.

**Table 3:** Follow-up final diagnosis for cases diagnosed as metastatic neoplasm and malignancy NOS by FNA.

Cytological Diagnosis	Total	Primary Site	No. of Cases
Metastatic neoplasm	75		
adenocarcinoma	68		
		Gastrointestinal	43
		Lung	11
		Pancreatic/biliary	4
		Prostate	1
		Ovary	1
		NOS	8
Squamouscellcarcinoma	7		
		Lung	3
		Nasopharyngeal	3
		Cervix	1
Malignancy NOS	258		
Carcinoma	142		
		Liver	74
		Gastrointestinal	49
		Pancreatic/biliary	5
		Lung	10
		Breast	2
		Nasopharyngeal	2
Adenocarcinoma	94		
		Liver	64
		Gastrointestinal	23
		Pancreatic/biliary	3
		Lung	3
		Ovary	1
Sarcoma	21		
		Liver	18
		Soft tissue	3
Carcinosarcoma	1		
		Liver	1

**Table 4:** Diagnostic outcome of different AFP levels.

	0–200	0–50	51–100	101–150	151–200
No. of patients	1756	466	355	377	558
(malignant/benign)	(1590/166)	(422/44)	(323/32)	(341/36)	(504/54)
True positive	1478	395	299	312	472
True negative	166	44	32	36	54
False positive	0	0	0	0	0
False negative	112	27	24	29	32
Sensitivity(%)	92.96%	93.6%	92.57%	91.5%	93.65%
Specificity(%)	100%	100%	100%	100%	100%
Positive predictive value(%)	100%	100%	100%	100%	100%
Negative predictive value(%)	59.71%	61.97%	57.14%	55.38%	62.79%
Overall accuracy(%)	93.62%	94.21%	93.24%	92.31%	94.27%

**Table 5:** Diagnostic outcome between the subgroups with tumor size<2 cm and ≥2 cm.

	<2 cm	≥2 cm	P value
No. of patients	149	1607	-
(malignant/benign)	133/16	1457/150	-
Sensitivity	93.23%	92.93%	0.896
Specificity	100%	100%	-
PPV	100%	100%	-
NPV	64%	59.29%	0.647

PPV, positive predictive value; NPV, negative predictive value.

### Hemorrhage

In this study, six cases of internal hemorrhage were diagnosed within 24 hours after FNA was performed. Most of the cases which suffered from hemorrhage after FNA occured in the early period, with two in 1980s and three in 1990s. Table 6 summarizes the characteristics of these cases. Although an intense effort was made to rescue the patients, the internal bleeding was fatal for three patients presenting with intraperitoneal hemorrhage. Subcapsular hemorrhage was detected in three cases, but did not cause death. These patients were treated with supportive and symptomatic care.

**Table 6:** Characteristics of hemorrhage.

Case Number	1	2	3	4	5	6
Age	39	48	64	51	47	52
Gender	Male	Male	male	Male	male	Male
AFP	23.3	5.9	78.49	139	11.33	20.12
Tumor type	HCC	HCC	HCC	HCC	HCC	HCC
Tumor size (cm*cm)	12.2*13.1	13.9*11.5	9*7.7	5.7*5.5	8.9*8.2	10.4*10.8
Tumor location	Subcapsular	Subcapsular	Subcapsular	Subcapsular	Subcapsular	Subcapsular
HBsAg	+	+	—	—	+	+
Cirrhosis	+	+	+	+	+	+
Prothrombase time(s)	10.9	11.4	12.0	12.7	13.1	13.3
Platelet count (10^9^/L)	78	124	185	67	96	135
Number of needle passes	1	1	2	2	2	1
Hemorrhage location	Intraperitoneal	Intraperitoneal	Intraperitoneal	Intraperitoneal	Intraperitoneal	IntraPeritoneal
Treatment outcome	dead	dead	dead	alive	alive	alive
Interval between onset and death(hour)	24	2	8	-	-	-

### Treatment and prognosis of implantation metastasis

The risk of needle tract metastasis implantation in this retrospective study was relatively low. Of all the patients who underwent FNA, only four cases (0.23%) of implantation metastasis were detected, and all of these cases had a history of liver cirrhosis. Two cases were detected on the thoracic wall, while the other two metastasized to the abdominal wall. The tumor size of the metastatic patients was relatively large with sizes ranging from: 8*10 cm, 11*12 cm, 16*15 cm, and 10*13 cm. The time for detection was 34, 118, 95, and 67 days respectively. Two cases received radical resection of the metastasis and two cases were performed with external radiation therapy. The survival time did not seem to be impacted by the metastasis, with 31, 11, 26 and 28 months respectively. Most likely, the patients died due to tumor progression and hepatic failure. It is worth mentioning that since 2007, after removal of the FNA needle during the procedure, manual pressure has been applied on the wound with an anhydrous alcohol cotton swab for at least 3 minutes. Since this addition, no post-procedural implantation metastases have been detected.

## Discussion

### Why advocate performing FNA in patients suspected of PLC with AFP ≤200 ng/ml?

The accuracy of cancer diagnosis is important since treatment regimens vary depending on cancer type and a false diagnosis can lead to unnecessary patient suffering. Over the last decade there has been a debate in the role of FNA in the detection of HCC. Advances in dynamic imaging techniques have increased the accuracy of HCC diagnosis in most nodules. Most of these dynamic imaging techniques are based on the vascular criteria. Computed tomography (CT) and magnetic resonance imaging (MRI) have a high sensitivity (55%–91%) and specificity (77%–96%) in diagnosing HCC [6]. However, there are several limitations to the assessment of HCC using only the vascular criteria. Under various circumstances, not all HCCs radiological imaging have typical “fast-in and fast-out” patterns, nor do they always present with significant AFP elevations. This can make the differential diagnosis of HCC from metastasized liver cancer and other tumors located on the liver difficult. For example, the enhancement pattern of small HCC depends on size and cellular differentiation, and tumor size less than 2 cm may have atypical enhancement. The diagnosis of HCC based on vascular pattern may overlook the hypovascular tumors. Conversely, 52% of small early arterial-enhancing lesions decrease in time and can be considered pseudolesions [7]. Meanwhile, with liver cirrhosis at baseline, a clinical differential diagnosis of multi-nodular cirrhotic nodules from highly differentialted malignant nodules is difficult to make. This leads to differentiating HCC from benign lesions commonly seen in cirrhosis or from secondary malignancies, which remains a challenge. Serum AFP used alone can be helpful if levels are markedly elevated, which occurs in fewer than half of all cases at time of diagnosis [5]. If based on radiological images and AFP levels alone, atypical hemangiomas with diameters less than 3 cm, metastatic tumors with necrosis, cystic degeneration or rich blood supply, inflammatory pseudotumors of the liver, and focal nodular hyperplasias, may lead to a false diagnosis, causing unneccessary surgical resection, liver transplantation, or other inappropriate treatments. In this study, there was no significant difference in the sensitivity, specificity, PPV and NPV according to P value between the subgroups with tumor size<2 cm and ≥2 cm, which showed the advantage of FNA in the diagnostic effectiveness in small hepatic lesions.

Pathological diagnosis can provide multiple advantages to patients searching for an appropriate treatment option for their suspected malignancy. FNA can provide a pathological sample for early diagnosis and data that can be utilized in medical research. Further, for patients suspicious of small HCC, especially those with atypical lesions on radiological imaging, FNA can mitigate patients' anxiety once a liver nodule has been detected. Detecting a liver nodule early can decrease costs that long-term imaging surveillance can accrue and provide patients with appropriate treatment options earlier.

### Analysis of low NPV

The sensitivity and specificity in our study was 92.96% and 100% respectively. The sensitivities of FNA for detecting malignancy in recent large research series have ranged from 83.3% to 97.5%, with specificities approaching or achieving 100% [8]. However, the NPV of our study was approximately only 60%. The reasons are analyzed as follows. Since Shanghai Cancer Center specializes in tumor treatment, most patients who present to the hospital are highly suspicious of malignancy, and only a relatively small number of patients have benign liver diseases. Other various factors may have influenced the results of FNA. For instance, patients with tumors located too close to the diaphragmatic surface and/or who failed to keep their breathing at a low rate during aspiration may have had compromised ultrasound images. This may have led to deviations along the fine needle tract which prevented obtaining an adequate sample. Clinically, it has been recognized as relatively difficult to diagnose highly differenciated HCC from regenerative nodules. Additionally, for multiple nodular cases with background liver cirrhosis, there is a possibility of aspirating non-malignant nodules such as liver cirrhosis nodules. Also, in the cases presenting with large tumors and cystic necrosis, the aspirated samples may have had liquefied material without malignant cells or may have failed to meet basic pathological investigation requirements.

### AFP levels and diagnostic outcomes

We analyzed the FNA results of tumors with different AFP levels ranging from <50 ng/ml, 50–100 ng/ml, 100–150 ng/ml, and 150–200 ng/ml. There were no significant differences seen in regards to sensitivity and NPV of FNA results among different AFP levels. Thus, according to our study results, we interpreted that AFP levels do not influence the results of US-FNA.

It is well-known that early detection of HCC not only increases patients' chances of receiving treatment, but also improves prognosis. The most commonly used confirmatory diagnostic modality of HCC is FNA. Although FNA is only a minimally invasive diagnostic approach, it still has serious side effects and can contribute to patient suffering. Since both AFP and FNA are commonly used diagnostic approaches for the final confirmatory diagnosis for PLC, it is important to evaluate the effectiveness and relationship of these two diagnostic methods to avoid unnecessary procedures and patient suffering. Unfortunately, there are limited studies focusing on the FNA value in patients with serum AFP ≤200 ng/ml. Most of the studies focus on either the prognosis of different AFP levels or the recurrence and side effects of FNA in relationship to tumor size. Based on 4,312 patients, this retrospective study was the largest population of patients with AFP ≤200 mg/l and suspected PLC, in which FNA has been performed. This may prove to be valuable for future clinical practice.

### Complications

As previously reported, biliary peritonitis is a serious complication of FNA [9], which fortunately, was not detected in any patients during the study. This may be related to our ability to avoid aspiration in cases where bile ducts are inevitably being punctured. The most significant complications observed in our department were hemorrhage and implantation metastasis.

### Normal or only mildly reduced coagulation parameters do not prevent bleeding complications

Risk of hemorrhage is not as controversial as implantation metastasis. A mortality rate of 0.018% was reported in a multi-institutional Italian series of 10,766 US-guided FNA biopsies [10]. All of the patients in this study who presented with post-FNA hemorrhage, also presented with liver cirrhosis and platelet counts above 50,000/µl. Further, no significant abnormalities in INR, coagulation profile, and/or prothrombin time (PT) were detected. Although the doctors' skills helped avoid puncturing major liver vessels, post-FNA hemorrhage still occurred. According to our clinical experience, hemorrhage is typically associated with severe cirrhosis or large superficial tumors not covered by normal liver parenchyma, rather than coagulation profiles and platelet counts. Similar to published literature, our data suggests that normal or only mildly reduced coagulation parameters do not prevent bleeding complications [11]–[13]. If the tumor is covered with enough liver parenchyma, it still can be punctured and without excess risk of hemorrhage, no matter the tumor size is more than 5 cm or not. Careful monitoring for post-FNA hemorrhage should be practiced for the tumors relatively large in size or in those that have relatively small amounts of normal liver tissue coverage. Meanwhile, before the procedure of FNA, the risk of bleeding should be evaluated in every patient. If the bleeding risk is high, imaging approaches should be used to diagnose the disease to avoid hemorrhage after FNA, although we advocate every patient who is suspected with PLC should undergo FNA.

### Post procedure monitoring and haemostatic agents are suggested for patients who underwent FNA

While not all reports on FNA acknowledge post-procedural hemorrhage, an acceptabley low incidence of hemorrhage does exist both theoretically and clinically. Thus, as routine practice after FNA, our department uses haemostatic agents. Until now, no prospective study has shown that using routine haemostatic agents decreases the risk of hemorrhage after FNA. It is not yet known if the use of haemostatic agents or remaining immobile for several hours following FNA increases a patient's risk of thrombosis. The incidence of hemorrhage in this retrospective study was very low with only 6 cases (3 major, 0.17% and 3 minor, 0.17%) detected in the 1,756 cases that underwent FNA. This may be due to the routine use of haemostatic agents after FNA. Meanwhile, most of the cases who suffered from hemorrhage after FNA occured in the early period, during which there were not many effective hemostatic agents. Comparatively, the newly emerged hemostatic agents may decrease the incidence of bleeding after FNA, which make this procedure much safer than that in the early period. Additionally, an increased risk of thrombosis was not observed. Thus, based on this study, routine use of haemostatic agents is suggested after FNA.

### Post-FNA monitoring should be carried out for at least 6 hours to minimize fatal complications

In all of the patients who experienced bleeding complications, including subcapsular and intraperitoneal hemorrhage, bleeding occurred within 6 hours after FNA. Thus, the experience from this study suggests that post-FNA monitoring should be carried out for at least 6 hours to minimize fatal complications. The patients that suffered from subcapsular hemorrhage were mainly treated with haemostatic agents, broad pressurizing belly bands, and blood volume expanders. The complication of subcapsular hemorrhaging resulted in no patient deaths.

For the three patients who experienced peritoneal hemorrhage, transcatheter arterial embolization was applied to two, while the other patient received a broad pressurizing belly band, blood transfusion, haemostatic agents, as well, as suppportive care. Unfortunately, peritoneal hemorrhage was fatal to all patients within 24 hours after detection. According to our study, the location of the hemorrhage site has a significant relationship with prognosis. Once peritoneal hemorrhage occurs after FNA, the condition will quickly become severe within a relatively short time period, making survival unlikely. Therefore, the patients complaining of severe abdominal pain after FNA, with signs of peritoneal irritation, significant haemoglobin decline, hemodynamic instability, and seroperitoneum detected on ultrasound investigation, should be treated for post-FNA hemorrhage immediately.

### Prognosis and treatment of implantation metastasis

Implantation metastasis has always been the major argument when deciding if FNA should be performed. Risk of implantation metastasis after FNA for malignancy, in general, is considered rare. An overall incidence of 0.13% of HCC with soft tissue metastasis was reported in one large study [14] where a total of 18,227 person-times of FNA or percutaneous ethanol injections were performed on HCC patients. Sliva et al. [15] in a systemic review and meta-analysis of eight observational studies found that the incidence of needle tract seeding after FNA varied greatly from 0–5.8%. However, FNA is not the only procedure that may lead to implantation metastasis. Other procedures, including: percutaneous ethanol injection, radio frequency abalation, and percutaneous transhepaticcholangial drainage, may all potentially induce implantation metastasis. Dong-Won Ahn, et al. [16] reported that two (0.13%) of the 1,549 patients who underwent PEI, four (0.12%) of the 3,391 who underwent FNAB, and one (0.66%) of the 152 patients who underwent PTCD for HCC, experienced needle tract seeding. In animal models, Ryd et al. [17] found that FNA can implant 10^3^–10^5^ cells along a single needle tract. Thus, theoretically, multi-site aspiration or multi-passes may lead to an increase in the amount of malignant cells at the implantation site and needle tract, and therefore increases the possibility of implantation metastasis. Our study verifies Ryd's findings. Among the four patients who presented with implantation metastasis after FNA, three had tumors larger than 10 cm in diameter. If the initial aspiration obtained majorly liquefied necrotic material, the pathologist considered the sample inadequate, and FNA was performed on another side of the lesion, which may have negatively influenced the pathological diagnosis. These resulted in repeated aspirations with a mean of four passes and at least two sites of aspiration among the four patients. Multiple aspiration sites and several passes may increase the chance of implantation metastasis. Thus, to reduce the possibility of implantation metastasis, we suggest that needle passes during FNA be reduced to the least number possible and the puncture should ideally be performed on a single site.

Meanwhile, the decline in implantation metastasis since 2007 may be an advantage resulting from manual compression on the puncture site with an alcohol cotton swab. Interestingly, the absolute alcohol can lead to liver cancer cell lysis and degeneration, which is often used for intra-tumor injections as a treatment option for HCC. Based on our experience, we suggest routine manual compression of an anhydrous alcohol swab on the needle site for at least three minutes in every FNA case to reduce the chance of implantation metastasis.

Despite the risk of implantation metastasis in FNA, however, surgical resection of the implantation site or radiological therapy may have a satisfactory treatment outcome. In the four cases of implantation metastasis in our study, two received regional radiological therapy (2500 cGy, 100 cGy per time) and two received radical excision. All of the cases had satisfactory treatment outcomes, and implantation tumors were eradicated in three cases, with no reoccurrence detected within the survival time. One case that received radiological therapy had a significant reduction in the implanted site's size and no significant progression was seen.

Additionally, since most cases in this study were unresectable, implantation metastasis didn't influence the prognosis of these patients. The patients who suffered died because of HCC progression and hepatic failure, not implantation metastasis itself. We suspect there were additional cases of implantation metastasis, which were undetected prior to death possibly due to the progression of the disease and the short survival time of PLC patients. Implantation metastasis does not have the same importance and influence on prognosis as metastasis has on other organs such as lung, kidney, adrenal gland, bone, etc.

However, we do not suggest patients who meet clinical diagnosis criteria and who are eligible for radical resection or liver transplantation receive FNA since the risk of needle track metastasis does exist, which may lead to unradical tumor excision.

## Conclusion

Based on the large sample of patient characteristics in this retrospective study, we conclude that patients with PLC, especially those who present with AFP ≤200 mg/ml, should undergo FNA since a clinical diagnosis cannot be made based on serum AFP and imaging investigations alone. If negative results are obtained by FNA, it still could be HCC and repeated FNA procedure may be needed if highly suspicious of HCC on imaging study. US-FNA may be considered a relatively safe procedure with minimal side effects, however, the risk of fatal internal hemorrhage and needle tract implantation metastasis should not be overlooked. Therefore, we suggest that US-FNA be performed in hospitalized patients who will receive 6 hours of routine monitoring after FNA. To our knowledge, this is the first and largest study concerning the relationship and predictive value of FNA in patients with PLC presenting with serum AFP ≤200 mg/l. Thus, we conclude that FNA is a necessary procedure for disease diagnosis since the benefits outweigh potential complications due to superiority in sensitivity, specificity, and overall accuracy.
